# Cluster randomised feasibility trial of PRISM: the PRimary Care Individual Social Norms MSK Data Dashboard to support first contact physiotherapy management of musculoskeletal patients in primary care

**DOI:** 10.1136/bmjopen-2026-118099

**Published:** 2026-07-21

**Authors:** Emma Dunphy, Louise Marston, Rachael Hunter, Jonathan C Hill, Anne Marie Downey, Sharon Forsyth, Chidimma Nwankwo, Irwin Nazareth

**Affiliations:** 1 Department of Primary Care and Population Health, Upper Third Floor UCL Medical School (Royal Free Campus), University College London, London, UK; 2 School of Allied Health Professions and Pharmacy, Keele University, Newcastle-under-Lyme, UK

**Keywords:** Primary Health Care, Quality Improvement, Musculoskeletal disorders, Physical Therapy Modalities, Behavior

## Abstract

**Introduction:**

Musculoskeletal (MSK) conditions affect over 20 million people in the UK and account for one in seven General Practitioner (GP) consultations. To address capacity challenges, First Contact Physiotherapists (FCPs) now manage MSK patients in primary care. However, FCPs working in isolation from physiotherapy teams exhibit broad variation in decision-making that could pose risks to care quality. PRimary Care Individual Social Norms MSK Data Dashboard (PRISM) is a clinician-facing behaviour change digital dashboard that provides individualised social norms feedback such as, ‘you are in the top 10% of those referring to secondary care’ or ‘in the bottom 10% of those referring to social prescribing’. It aims to nudge FCPs towards evidence-based norms, including more effective and cost-efficient treatments, thereby reducing unwarranted variation in care and improving patient outcomes. Social norms interventions have been shown to positively influence clinician behaviour, but their application in MSK services remains unexplored.

**Aim:**

To determine the feasibility of evaluating PRISM’s effectiveness and cost-effectiveness in a future clinical trial.

**Methods and analysis:**

A pragmatic feasibility cluster randomised controlled trial will be conducted across four UK primary care sites with FCP services. Sites will be allocated 1:1 to intervention (PRISM dashboard, guidebook and structured clinical supervision) or control (usual care). Data on clinical decision-making will be collected monthly and anonymised for dashboard generation and compared to an existing dataset of over a million FCP consultations. Patients seen by FCPs will be invited to provide patient-reported outcome measures and experience measures. Feasibility outcomes include recruitment and retention of sites, FCPs and patients; dashboard engagement and completeness of data collection.

The primary outcome is recruitment feasibility. Secondary outcomes include retention, dashboard usage, patient-rated outcome measure/experience measure (patient-rated outcome measures/patient-rated experience measures) completion and supervision engagement. Descriptive analyses will summarise feasibility metrics and missing data. Health economic and carbon reduction data collection will also be assessed for future trial planning.

**Ethics:**

IRAS number 361 359. REC reference: 25/EM/0256, Approved: 10 December 2025 based on PRISM Protocol V.9. Dated 25 November 2025.

**Trial registration number:**

NCT07112508.

**Dissemination:**

Findings from the PRISM feasibility trial will be disseminated through peer-reviewed publications, conference presentations and stakeholder engagement. Results will also be shared with participating sites, patient and public involvement representatives and professional bodies such as the Chartered Society of Physiotherapy to inform clinical practice and policy. Additionally, lay summaries will be produced for patients and the public and digital channels will be used to maximise reach.

**STRENGTH AND LIMITATIONS OF THIS STUDY**

- This protocol uses a cluster randomised feasibility design embedded within routine primary care services, enabling robust testing under real-world conditions.
- The PRISM trial structure is pragmatic and promotes First Contact Physiotherapist (FCP) data collection beyond the research by both using established routine data collection and facilitating new data collection where needed.
- Heterogeneity of services may lead to inconsistency across some metrics.
- Clinician data are self-reported to avoid significant operational barriers that FCP services experience in primary care. This could lead to errors in data collection; however, audit will be used to ensure accuracy of monthly data extraction.

## Introduction

 Over 20 million people in the UK live with a musculoskeletal (MSK) condition, accounting for one in seven GP consultations.^1^ The Fuller Stocktake Report from National Health Service (NHS) England described services as ‘stretched beyond capacity’ with ‘signs of genuine and growing discontent’.^2^ The NHS, in keeping with the international trend to include non-GP clinicians in the Primary Care workforce,^3 4^ has incorporated pharmacists, physiotherapists, practice nurses and physician associates within the primary care workforce to address the challenges of capacity and demand.^5–7^

In the UK, patients with MSK problems consult a First Contact Practitioner (FCP) based in primary care instead of their GP, improving patient access to MSK management and freeing GP capacity.^8^ The FCP service in the UK, Sweden and Canada was found to be acceptable and effective in national evaluations.^5 7–10^

The Chartered Society of Physiotherapy (CSP) and Health Education England called for FCPs to be ‘Advanced Practitioners’ (APs),^11 12^ a certification conferred on those with particular expertise. This was deemed necessary to meet the level of risk associated with having a clinical caseload of patients who have not been screened by a doctor.^13^ However, the pool of APs is limited and increasingly less experienced FCPs have been recruited into these posts.

FCPs in GP practices work alone^9^ and lack the peer support or oversight of a typical physiotherapy department. Data suggest that less experienced FCPs may have different patterns of behaviour than their more senior colleagues. Clinical audits of FCP decision-making in over 2000 patients, from the Midlands and London, show distinct differences in decision-making where Band 7 FCPs were almost twice as likely as Band 8 colleagues to send the patient to the GP for a second opinion^14^ and had different behaviours in ordering imaging and referring patients onwards to other services. The appropriate use of skills such as imaging requests and onward referrals should be used with consistency and in step with evidence-based guidance. New methods are needed to identify variation in care at an individual level and support clinicians to move toward best practice.

Evidence suggests supervision and feedback in clinical contexts can improve care.^15^ Specifically, that clinical supervision is an effective aspect of professional training and development that can improve evidence-based practice.^16 17^ It has been shown to support the effectiveness of care^18^ and reduce burnout and stress.^19^ A Cochrane review highlighted the importance of individual tailored feedback in supervision for learning and improving clinical standards.^20^

There have been calls for better use of data related to clinician performance in clinical supervision. Data on patient outcomes or experience or clinician behaviour would have the potential to highlight variations in care by providing more formal and structured feedback on decision-making and quality of care.^21 22^ Highlighting variations and outliers in clinical behaviour offers clinicians a unique chance to recognise when their decision-making has deviated from the expected norms for their role. Presenting data feedback about an individual’s position in relation to their peers is called ‘social norms’ feedback and can be used to identify outliers in clinical decision-making and nudge behaviour change. In behaviour change theory, social norms refer to the unwritten rules and expectations about how individuals should behave in specific situations.^15 23^ These norms are shaped by the collective perceptions and behaviours of a group, influencing what is considered appropriate and expected.^23^ A recent systematic review and meta-analysis showed that social norms interventions are effective in ‘changing health care practitioner behaviours and improving patient outcomes’.^15^ They nudge clinician behaviour towards desired standards and reduce variation in care.^24 25^ The potential of social norms interventions remains unexplored in MSK services in Primary Care.

This research introduces PRISM: the PRimary Care Individual Social Norms MSK Data Dashboard. It is a monthly digital dashboard based on up-to-date clinical activity data, that provides clinicians with personalised feedback on their practice by benchmarking it against aggregated social norms of FCPs. The social norms for FCP refer to the appropriate and expected behaviour of FCPs based on the behaviour of the majority and professional standards. For example, ‘You are in the top 10% of those ordering MRI or referring to orthopaedics’ or ‘you are in the bottom 10% of those referring to social prescribing or health and wellbeing services’. The dashboard is shared with individual FCPs and their clinical supervisors and adjustable for demographics such as Index of Multiple Deprivation and population characteristics. It will aim to report on the NHS England, ‘Quality Safety and Effectiveness’ measures for MSK Primary Care^26 27^ and other measures agreed by stakeholders in a related qualitative work package for this study.

With the support of the CSP and a network of improvement-focused FCP services across the country, we have worked to create a dataset comprising over 1 000 000 FCP contacts that we can analyse. The datasets are not uniform but have a core set of common metrics drawn from guidance from CSP and physiotherapy leaders.^26 28 29^ The methods of data collection also vary, including data reported from electronic patient records and data collected through purpose-built forms. If the datapoints capture the activity in FCP service, then it can be used for individual social norms feedback to FCPs in the PRISM study.

Although service level data already exist in many areas and enable knowledge at a provider level, they do not address variation in clinician performance or provide a mechanism for individual clinicians to reflect on their decision-making and change behaviour. The PRISM research has formed a collaboration with industry partner Visualising Underlying Indicators and Trends (VUIT) Data Labs to develop an interactive individual clinician data dashboard that compares an individual FCP’s activity against the ‘norms’ of the dataset.

### Research question

Is it feasible to recruit and retain patients and FCPs to the PRISM study? Is there sufficient uptake of the PRISM intervention? Is it feasible to collect patient-rated outcomes and experience measures and clinical activity, cost and carbon data for a future clinical trial?

### Aim and objectives

#### Aim

To determine the feasibility of evaluating PRISM’s effectiveness and cost-effectiveness in a future clinical trial.

#### Objectives

1. To assess the feasibility of recruiting and retaining participants and FCP teams to the trial.
2. To assess the feasibility of collecting patient-rated outcome measures (PROMs) and patient-rated experience measures (PREMs) from FCP patients and outcomes from FCPs.
3. To assess the implementation of FCP data collection in both arms of the study.
4. To assess the engagement of FCPs or their supervisors with the intervention.
5. To inform the protocol for a fully powered randomised controlled trial (RCT) to determine the clinical and cost-effectiveness of using the PRISM Dashboard and Guidance Document.

## Methods

### Study design

This is a pragmatic feasibility cluster randomised controlled trial with a related qualitative work package not described here. The setting will be UK general practices that have an FCP service. There is an ideal opportunity to evaluate the PRISM intervention including a social norms dashboard and training guidebook within this existing national network.

### The PRISM intervention and control

The intervention is described in line with the TiDieR template for intervention description (online supplemental appendix 1).^30^ There are four parts to the intervention.

**Figure 1:**
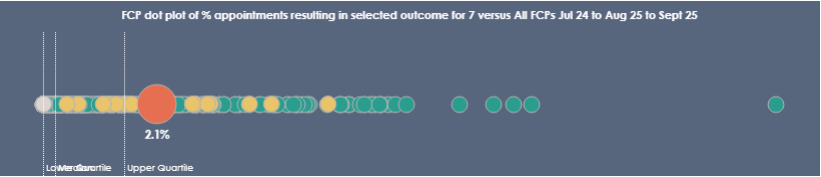
A snapshot from the dashboard shows an FCP whose MRI numbers are outside the upper quartile ‘social norms’ of FCPs referring for MRI. Green are all FCPs, yellow is FCPs from the same provider and red is the individual. Floating the mouse over any dots gives the proportion of patients referred by any FCP for investigations and the numerator and denominator. FCP, First Contact Physiotherapy.

**Figure 2:**
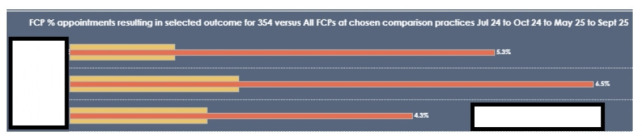
This shows all the practices that this FCP works at vs other FCPs at those practices. This FCP is ordering significantly more MRIs than FCP colleagues at the same practices. Floating the mouse over the bars gives the proportion of patients and the number of appointments and number of appointments where MRI was an outcome. FCP, First Contact Physiotherapy.

**Figure 3:**
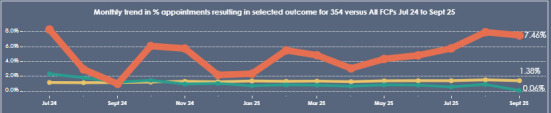
This graph shows MRI trends of all FCPs (green), FCPs from the same provider (yellow) and individual FCP (red) over the time we have data for this FCP. FCP, First Contact Physiotherapy.

1. Data collection
  FCP clinical assessment and patient management metrics (listed fully as secondary outcomes in online supplemental appendix 2) will be collected from FCP clinics in the intervention and control arms using their usual data collection practices. This includes data entered directly on Egton Medical Information Systems (EMIS) and SystmOne or data that are entered on manual forms and spreadsheets by FCPs. In some cases, these data are collected routinely for service level data analysis. Some clinics use a template that was developed by the Keele MIDAS (Multi-level Integrated Data for musculoskeletal health intelligence and ActionS) team in collaboration with the CSPs and NHS collaborators.^26 28 29^ The template is used by FCPs at the end of each consultation and takes a maximum of 1 min to complete. It is a summary of the consultation, with each tick-box being linked to the relevant Systemized Nomenclature of Medicine—Clinical Terms codes (SNOMED-CT) to enable clean data extraction from the consultation record. This fully anonymised data will be transferred securely on a monthly basis to the University College London (UCL) Data Safe Haven where the lead researcher will prepare it to the correct format before sharing it with our dashboard developer VUIT Data Labs.
2. The PRISM dashboard
  The PRISM dashboard will be developed with commercial partners VUIT Data Labs. FCPs and their supervisors in the intervention group will receive the password-protected dashboard monthly via email link. The dashboard will feature visual representations of their clinical decision-making, benchmarked against the aggregated data of all FCPs which is presented as the ‘social norm’. The data will be presented per metric and in relevant groups; for example, an FCP proportion of patients referred for investigations will be shown as ranked dot plot with median and IQRs to visualise the distribution of data and identify where the individual’s behaviour sits within the social norms of a whole group (figures 1–3). The dashboard explores proportions and can filter to compare areas with similar levels of deprivation, unemployment, etc.
3. The guidebook
  FCP participants will be provided with a detailed guidebook. It will contain sections on how to interpret the data, the evidence base that informed its development and a logic model of how it can lead to better care. The guidebook will support the FCP and their supervisor while they review the dashboard in clinical supervision.
4. Structured clinical supervision
  The FCP and their supervisor will review the dashboard in monthly clinical supervision for 1 hour. The data will inform knowledge of the complexity of the caseload, outlier occurrences, common challenges such as decisions around imaging or investigations and referrals onwards.

Control: Clinicians in the control group will continue treatment as usual over the trial period; they will engage in supervision, submit data and complete outcomes but they will have no feedback until the trial is over.

### Participants

#### Site eligibility

First Contact Physiotherapy (FCP) sites who express an interest will be given the Site Information Sheet and assessed in terms of eligibility. Sites refer to the FCP team employer and may be NHS Trusts, private providers or GP practices. Eligibility will be determined by the inclusion and exclusion criteria for sites, as below. Data sharing agreements will be in place, and the research team will perform an audit of data collection processes.

#### Site recruitment

Site recruitment will be obtained via the FCP service lead. The FCP service lead will be given the Site Information Sheet and provided with an opportunity to ask questions. Sites will be given 2 weeks to decide whether they are able to take part. The 2-week period reflects the fact that multiple persons may need to be consulted before a decision to consent is made. It will be recorded in the trial record when the Site information sheet is given. Site agreement will ultimately be taken by the CI.

#### FCP eligibility and consent

Once the site agreement is in place, individual FCPs will be offered the FCP information sheet and an opportunity to ask questions and 24 hours to consider before consent will be taken (FCP Consent form online supplemental document 1). They are consenting to have their clinical activity data collected to inform the PRISM dashboard. They are consenting to viewing the dashboard to understand their position within national and local norms. They are consenting to reviewing the dashboard in their monthly clinical supervision and to completing a log and a supervision outcome measure. Those who do not consent will be invited to give a reason but are not required to. If they give a reason, it will be recorded as part of the trial record.^31^

#### Participant identification centres

Individual FCP eligibility will be assessed according to the inclusion and exclusion criteria below. If FCPs agree to participate, the GP practice in which they work will be set up as Participant Identification Centres (PICs). GP Practices will be approached with a GP Practice Information Sheet that explains the study. They will need to agree to two activities:

1. Running a weekly search of FCP patients, supported by the Research Design Network.
2. Sending a standardised message to the identified patients inviting them to participate in the study.

GP practices will be paid to conduct these activities. This process is supported by the RDN who will work with GP practices to undertake weekly searches of the primary care medical records to identify patients who were seen by an FCP. Patients identified in the search will be sent an invitation text or email from the GP practice followed by a reminder text that tells them their practice is involved in this research and invites them to participate. It will include a link to the participant information leaflet, data privacy statement and consent forms (Patient Consent form online supplemental document 2). Should the patient consent, they will be registered as part of the study and a link to an online REDCap survey will be sent. All patients will receive treatment as usual. The schedule of events for all participants is included in online supplemental appendix 3.

#### Inclusion and exclusion criteria for sites and individual FCPs

Site inclusion criteria

- FCPs must have regular clinical supervision by a more experienced physiotherapist.
- Healthcare Professionals Council registered Physiotherapists.
- Provide physiotherapy to NHS patients.
- Able to send monthly data uploads to the UCL Data Safe Haven to be added to the PRISM dashboard.

Patient inclusion criteria

- 18 years of age and above.
- Registered with a GP practice that is a PIC for this study.
- Consented to provide outcome measures as part of the trial process.

Site exclusion criteria

- No clinical supervision by a more experienced physiotherapist available currently or in practice for the FCPs.
- Community service physiotherapy.
- Unable to send data to the UCL Data Safe Haven monthly.

Patient exclusion criteria

- Non-MSK problem.
- Insufficient level of English understanding and expression to allow independent completion of assessment instruments.
- Lacking capacity or unwilling to consent.
- Patients who attended FCP but were not treated as they were found to have been an inappropriate referral.

#### Patient consent

Patients who see an FCP during the period of the study will be sent a text by the practice with a link to the study details including the PIS and consent forms and the opportunity to request a phone/video call to support the e-consent process. Consent will be obtained digitally and if requested they can be supported to complete the digital consent over the phone. The process will be overseen by a GCP-accredited researcher, either the CI or a suitably qualified and experienced person. For individual consent, a minimum of 24 hours for consideration will be given. The participant will have the opportunity to ask questions. It will be recorded in the case notes when the participant information sheet has been given to the participant. The Investigator will explain that participants are under no obligation to enter the trial and that they can withdraw at any time during the trial, without having to give a reason.

A copy of the signed informed consent form will be given to the participant. The original signed form will be retained in the trial file at site and a copy placed in the case notes.

#### Randomisation

Sites will be randomised to the intervention or control. The randomised allocations will be communicated by a data manager. The randomisation list will be compiled by a person not part of the study or the randomisation programme will be written by a statistician who is part of the study but the seed is changed by a data manager (so that the statistician does not know the allocation). It will be set up, tested and validated following PRImary care and Mental hEalth NeTworK (PRIMENT SOPs). A statistician will computer-generate the randomisation sequence and ensure concealment by providing each place with an anonymised code. Allocation will be shared with relevant study team members but not with the statistician or health economist doing the analysis. It was decided to randomise by site rather than by practice or primary care network as we thought the risk of intervention contamination between the intervention and control group was too great if they were within the same clinical teams.

#### Trial duration

The expected duration of the trial is 7 months for sites/FCPs from baseline to 6 months follow-up, allowing for data collection to begin 1 month before baseline outcomes are taken. The duration of the trial for patients is 3 months from baseline to final follow-up at 3 months. The end of trial is the last follow-up completion of the last participating FCP patient and FCP.

#### Serious adverse events

Although this feasibility trial is low risk and no adverse events are expected, we will proceed to determine the feasibility of collecting Serious Adverse Event data from participating sites. Collection, recording and reporting of adverse events (including serious and non-serious events and reactions) to the sponsor will be completed via a proforma provided to FCPs as part of the FCP site file and according to the clinical trials unit standard operating procedures.

### Data collection and data analysis

#### Data collection

Patients will be texted by their GP practice about the study. If they click the link to participate, patients will submit demographic details (name, age, ethnicity) which we will use for research analysis and complete outcome and experience measures (PROMs and PREMs) digitally, via a link to REDCap. Follow-up measures will be sent by text after 3 months.

FCPs and supervisors will keep digital logs of supervision sessions and engagement with the dashboard. This will also be recorded on REDCap and a link will be emailed to them on a monthly basis. PRISM dashboard usage (logins) will also be recorded. Trial data are stored on a secure server in the Clinical Trials Unit behind the University College London firewall.

#### Outcomes

The primary outcome for feasibility is recruitment defined by the ability to recruit FCPs and patients to the PRISM study. Secondary outcomes are detailed in table 1. Clinical behaviour metrics may vary based on the data collected by different services as part of their current data collection practice. A full list of clinical behaviour metrics is also listed in online supplemental appendix 2.

**Table 1:** Primary and secondary outcomes for PRISM feasibility

Category	Outcome	Details/metrics	Timepoints
Primary outcome	Recruitment	Number of participants recruited	Baseline
Feasibility outcomes	Retention Data collection engagement Dashboard engagement Patient outcomes collection Clinician outcomes collection Supervision log FCP characteristics Missing data	FCPs and patients retained Number of FCP encounters, completeness and characteristics of data collection Logins (FCP/supervisor), time spent, pages visited Clinical activity data, PROMs (EQ-5D-5L, MSK-HQ, WPIA, global change), PREMs (GP Communication Scale of GPAQ)^42^ Manchester Scale of Supervision^43^ Monthly entries by FCP and supervisor Years qualified, band, WTE%, appointment length Frequency and %	3FU, 6FU (FCPs); 3FU (pts) Monthly Monthly Baseline, 3FU Baseline, 3FU, 6FU Monthly Baseline Baseline, 3FU (patients); Baseline, 3FU, 6FU (clinicians)
Clinical decision-making: investigations	MRI requests, X-ray, US, CT, bloods Referrals: social prescribing, health and well-being, vocational services, FitNote Referrals: specialist Physio/MSK/ESP MCATS, Podiatry, OT, Falls Referrals: Neuro, Ortho, Rheum Back to GP for medical reason, GP for prescription, GP for injection, GP for referral	Proportion of all patients	Monthly
Social norms outcomes	Individual outliers Team outliers	Proportion of outlier occurrences (≥20%) Overall outlier occurrence	Monthly
Supervision outcomes	Supervision log MCSS-26^26^	Participation and dashboard/guidebook use Manchester Scale of Clinical Supervision	Monthly Baseline, 3FU, 6FU
Patient outcomes	PREM PROM Pain site	GP Communication Scale EQ-5D-5L, Musculoskeletal Health Questionnaire,^44^ modified WPAI,^45^ Global Percieved Effect^46^ Body site	Baseline Baseline, 3FU Baseline, 3FU
Health economic outcomes	EQ-5D-5L Cost per intervention	Health-related quality of life Imaging, referrals, platform, training, time costs	Baseline, 3FU 6FU
Carbon reduction outcomes	CO_2_ emissions	MRI, X-ray, ultrasound, equipment, consumables, waste, mobility, infrastructure	6FU

EQ-5D-5L, EuroQol 5-Dimension 5-Level; ESP, Extended Scope Practitioner; FCP, First Contact Physiotherapist; FU, follow up; GPAQ, Global Physical Activity Questionnaire; MCATS, Musculoskeletal Clinical Assessment and Treatment Service; MCSS-26, Manchester Clinical Supervision Scale (26-item version); MSk, musculoskeletal; PREM, patient-rated experience measure; PROM, patient-rated outcome measure; WPIA, Work Productivity and Activity Impairment.

#### Sample size

We will recruit from four sites with approximately 8–12 physiotherapists per site. Primary care areas will be allocated on a 1:1 basis. Four sites have been selected as these are the numbers that are feasible to work within the available timeframe.

For patient participants: It is expected that eight physiotherapists per site will see at least 10 patients per week. This is 80 patients per site per week and 320 patients per week across four sites. Over 3 months this will mean 3840 patients will be invited to take part in the study. If 5% respond we will have 192 patients. If 10% respond 384 patients

An estimate of the recruitment period for the trial is 3 months calculated based on the expected number of eligible sites and the time taken to assess against the inclusion/exclusion criteria. We aim to recruit 4 sites in 4 weeks.

#### Statistical analysis

This protocol is reported in line with the Standard Protocol Items: Recommendations for Interventional Trials (SPIRIT) guidelines for reporting outcomes in trial protocols.^32^ It is not possible to blind FCPs to the intervention arm. Patients will be blinded to the intervention arm. The statistician and health economist will be blinded to the randomisation in line with clinical trials unit protocols.

Analysis will be done using intention-to-treat principles based on clusters. Categorical measures will be summarised using frequencies and percentages. Continuous measures will be summarised using means and SD or medians and IQRs depending on distribution. All outcome measures will be summarised separately by study arm. Potential therapist effect will be assessed using the intraclass correlation coefficient. The precision of estimates will be assessed using 95% CIs. We will look at missing data on key variables in the primary or secondary outcomes as this is a feasibility outcome.

#### Economic evaluation

The analysis will focus on the feasibility of collecting data that would inform an economic evaluation in a full trial. We will also assess the feasibility of calculating the cost of the new dashboard in relation to any additional training or delivery resources or additional physiotherapy time will also be calculated. The feasibility of collecting Quality Adjusted Life Years (QALYs) over 6 months will be calculated from patient responses to questionnaires. We will report rates of missing data, mean utility scores and SD at each follow-up time point. QALYs are used in determining the additional value of a new healthcare intervention compared with current practice.^33 34^ The second component will be the feasibility of collecting resource use data to apply published unit costs to.^35 36^ Descriptive statistics will be reported for each resource per physiotherapy intervention. The average cost per patient will be calculated to evaluate any potential cost-savings from the new dashboard as a result in changes in decision-making practice.

#### Carbon reduction analysis

This analysis will sit alongside the health economic analysis to measure CO_2_ emissions and ‘carbon unit costs’.^36^ The NHS and National Institute for Health and Care Research (NIHR) have a strategy for carbon reduction.^37 38^ Research has demonstrated methods for scalable carbon reduction in Primary Care^39^ CO_2_ emission measurements for commonly used healthcare modes such as MRI or X-ray are available.^40^ Recent analysis of MSK workflow in primary care has mapped carbon-related costs using a cost and carbon calculator which will be useful for this study.^41^ This research will aim to identify the feasibility of collecting the relevant data for carbon reduction analysis in a future trial that focuses on FCP workflow.

### Trial management and PPI

#### Trial Management Group

The Trial Management Group (TMG) will include the Chief Investigator, trial manager, health economist, data manager and statistician. The TMG will be responsible for overseeing the trial. The group will meet four times during the study and will send updates to PIs at each site. We will also have a Trial Steering Committee (TSC), the role of which is to provide overall supervision of the trial. The TSC will also take on the function of data monitoring committee as this feasibility trial does not require a separate data monitoring committee. The TSC will include patient and public involvement (PPI) members. consider relevant information, recommend any appropriate amendments/actions for the trial as necessary.

#### Patient and public involvement

PPI representatives engaged via Healthwatch and UCL were actively engaged from the outset, contributing to the design of the research, the development of the intervention and the creation of participant-facing materials. Healthwatch PPI members provided valuable insights into patient priorities, acceptability of recruitment and consent. UCL-based PPI contributors worked closely with the research team to create patient facing documentation and to refine the dashboard and guidebook, ensuring these tools were accessible and relevant to patients’ needs. PPI representatives also participated in the qualitative work package that informed the intervention’s content and structure. Throughout the trial, a PPI member will sit on the Trial Management Group, where they help oversee trial conduct, review recruitment and safety data, and ensure that patient and public perspectives remain integral to decision-making and dissemination. This collaborative approach ensures the study remains grounded in the experiences and priorities of those it is designed to benefit.

### Ethics and dissemination

To maximise reach and impact, dissemination will adopt a multi-modal approach. Alongside publication in high-impact, open-access journals and presentations at national and international conferences, we will leverage digital platforms to engage diverse stakeholders. Infographics summarising key findings will be hosted on institutional websites and shared via professional networks such as the CSP and FCP and digital forums. Social media campaigns using concise visual narratives will target clinicians and policymakers, amplifying engagement through LinkedIn and X ensuring PRISM’s insights inform practice and policy at scale. PPI partners will co-create accessible summaries to ensure findings resonate with service users and community groups.

## Supplementary material

10.1136/bmjopen-2026-118099 online supplemental file 1

10.1136/bmjopen-2026-118099 online supplemental file 2

10.1136/bmjopen-2026-118099 online supplemental file 3

10.1136/bmjopen-2026-118099 online supplemental file 4

10.1136/bmjopen-2026-118099 online supplemental file 5
