# Supplementary material for: Cluster randomised feasibility trial of PRISM: the PRimary Care Individual Social Norms MSK Data Dashboard to support first contact physiotherapy management of musculoskeletal patients in primary care
Source: BMJ Open. 2026 Jul 21;16(7):e118099. doi: 10.1136/bmjopen-2026-118099 (PMC13404854; doi:10.1136/bmjopen-2026-118099)
Supplement: online supplemental file 1 [file bmjopen-16-7-s001.docx]

Appendix 1: Tidier template

| **TIDieR Item** | **Description** |
| --- | --- |
| 1. Brief Name | PRISM: The PRimary Care Individual Social Norms MSK Data Dashboard. |
| 2. Why (Rationale) | To address unwarranted variation in FCP clinical decision-making and promote evidence-based practice by providing individualised feedback on behaviour compared to peer norms, using social norms theory to nudge behaviour change. |
| 3. What (Materials) | - Data collection template (EMIS/SystmOne or equivalent, SNOMED-CT coded) - PRISM Dashboard (password-protected, visualising individual and group behaviour) - Guidebook (explains dashboard, evidence base, and interpretation) - Structured clinical supervision framework (using dashboard insights) |
| 4. What (Procedures) | - FCP clinical assessment and management data collected monthly from clinical systems or spreadsheets. - Data anonymised and uploaded to UCL Data Safe Haven. - Dashboard generated and distributed monthly to intervention group FCPs and supervisors. - Guidebook provided to support interpretation. - Dashboard reviewed in monthly clinical supervision sessions. |
| 5. Who Provides | - Dashboard and guidebook developed by UCL and Vuit Data Labs. VUIT Data Labs specialises in transforming complex population health and NHS data into actionable insights through interoperable datasets, advanced analytics, and interactive visualisation tools to improve patient outcomes and enable proactive, data-driven healthcare planning  - Delivered to FCPs and supervisors; supervision facilitated by clinical leads. FCPs and supervisors will be trained as part of study induction and given a guidebook to support use of the intervention. Weekly drop-ins will be available to support data understanding. |
| 6. How | - Digital delivery of dashboards will happen via secure email link. It will be viewed online by unique login.  - Guidebook distributed electronically or in print. - Supervision sessions scheduled within existing governance structures. |
| 7. Where | Primary care settings with FCP services across participating NHS sites. |
| 8. When and How Much | - Initial 4-week data collection phase. - Monthly dashboard updates during 6-month intervention period. - Ongoing monthly supervision aligned with organizational norms. |
| 9. Tailoring | The PRISM dashboard is tailored to each First Contact Practitioner by providing individualised, password-protected visual feedback on their clinical decision-making, benchmarked against aggregated peer norms and evidence-based standards, enabling personalised reflection and behaviour change during supervision. |
| 10. Modifications | The PRISM intervention is stable and ready to go to feasibility trial however, there will be opportunities to refine the intervention based on feedback captured in a related work package. |
| 11. How Well (Planned Fidelity) | Monitoring dashboard access and supervision records; adherence checks via trial database. |
| 12. How Well (Actual Fidelity) | To be assessed during feasibility trial through usage data and supervision logs. |
