# Supplementary material for: Cluster randomised feasibility trial of PRISM: the PRimary Care Individual Social Norms MSK Data Dashboard to support first contact physiotherapy management of musculoskeletal patients in primary care
Source: BMJ Open. 2026 Jul 21;16(7):e118099. doi: 10.1136/bmjopen-2026-118099 (PMC13404854; doi:10.1136/bmjopen-2026-118099)
Supplement: online supplemental file 2 [file bmjopen-16-7-s002.docx]

Appendix 2 :

| APPENDIX 2 – List of FCP metrics that may be collected by services and reported on in the data |  |
| --- | --- |
| **Category** | **Service Type** |
| **Rehab/Therapy Focused** | **Physiotherapy** |
|  | **Community Rehab Physiotherapy** |
|  | **Occupational Therapy** |
|  | **Podiatry** |
|  | **Dietician** |
|  | **Pain Service Community** |
|  | **IAPT/Mental Health Professional** |
|  | **Pharmacy** |
|  | **Nurse** |
|  | **Paramedic** |
|  | **Referral to Community Exercise Scheme** |
|  | **Falls/Frailty** |
| **Social & Community Support** | **Social Prescribing** |
|  | **Health and Wellbeing Practitioner** |
|  | **Employment Support Services** |
|  | **National Diabetes Prevention Programme** |
|  | **Smoking Cessation** |
|  | **Weight Management Services** |
|  | **Personalised Care Plan** |
|  | **Shared Decision-Making Tools** |
| **Medical Escalation & GP Support** | **Refer to GP for Medical Escalation** |
|  | **Refer to GP for Medication** |
|  | **Refer back to GP for Diagnostics** |
|  | **Refer back to GP for Onward Referral** |
|  | **Refer back to GP for FitNote** |
|  | **Indirect Support from GP (Safeguarding)** |
|  | **Indirect Support from GP (Complex Case)** |
| **Specialist Referrals** | **Orthopaedics** |
|  | **Neurology** |
|  | **Rheumatology** |
|  | **Advice and Guidance** |
|  | **A&E** |
|  | **Urgent Orthopaedics** |
|  | **Urgent A&E** |
|  | **Urgent Rheumatology** |
|  | **Urgent Neurology** |
|  | **Pain Service Secondary Care** |
|  | **Women’s Health / Pelvic Pain** |
| **Diagnostics** | **Dexa Scan** |
|  | **Nerve Conduction Study** |
|  | **X-ray** |
|  | **Ultrasound** |
|  | **MRI** |
|  | **CT Scans** |
|  | **Blood Tests** |
|  | **Single Point of Access** |
| **Self-Management & Education** | **Physical Activity** |
|  | **Self-Management Support** |
|  | **Digital Self-Management Support** |
|  | **% with OA and RA with HEP** |
|  | **Understanding / Confidence** |
|  | **Red Flag Data Captured** |
|  | **Education Given** |
|  | **CARE** |
|  | **Patient Activation Measure** |
| **Medication & Pain Management** | **NSAIDs (Topical)** |
|  | **NSAIDs** |
|  | **Opioids** |
|  | **Neuropathic Pain** |
|  | **Biphosphonates** |
|  | **Injections** |
|  | **OTC Medication Advice** |
|  | **Antidepressants** |
|  | **Deprescribing** |
| **Patient Experience & Outcomes** | **Physical Function** |
|  | **Wide Disability** |
|  | **Emotional Wellbeing** |
|  | **Impact on Work** |
|  | **Work Absence Productivity** |
|  | **Fit Note** |
|  | **FCP Follow-Up** |
|  | **Patient Experience** |
|  | **Vocational Advice** |
