# Supplementary material for: Cluster randomised feasibility trial of PRISM: the PRimary Care Individual Social Norms MSK Data Dashboard to support first contact physiotherapy management of musculoskeletal patients in primary care
Source: BMJ Open. 2026 Jul 21;16(7):e118099. doi: 10.1136/bmjopen-2026-118099 (PMC13404854; doi:10.1136/bmjopen-2026-118099)
Supplement: online supplemental file 3 [file bmjopen-16-7-s003.docx]

Appendix 3

Schedule of assessments for patient and for sites

| **Timepoint** | | **Assessment** | **Who Performs It** | **Mode** |
| --- | --- | --- | --- | --- |
| Screening | | Eligibility checks via search of Electronic Patient Record | RDN + GP Practice | Electronic |
| Baseline | PIS including opportunity for questions | | Research team (GCP-accredited) | Digital/Phone |
| Baseline | | Eligibility check by patient and Informed Consent |  |  |
| Baseline | | Patient Outcomes and Experience Measures (10.2) | Participant | Digital/Phone |
| 3 Months | | Follow up Patient Outcome measures | Participant | Digital/Phone |

| **Timepoint** | **Assessment / Activity** | **Who Performs It** | **Mode** |
| --- | --- | --- | --- |
| Pre-trial | Site eligibility and consent | CI / Sponsor | In-person/Email |
| Pre-trial | Individual FCP consent | CI/Sponsor | In person/email |
| Monthly from first baseline -1 month | Data upload to UCL Data Safe Haven | NHS Site Team | Secure upload |
| Monthly | Dashboard delivery (intervention arm only) | PRISM Team | Email link |
| Monthly | Structured clinical supervision using dashboard + guidebook | FCP + Supervisor | In-person/Remote |
| Monthly | Supervision log completion | FCP | Digital |
| Baseline | Manchester Clinical Supervision Scale | FCP | Digital |
| 3 months | Manchester Clinical Supervision Scale follow up | FCP | Digital |
| 6 months | Final Manchester Clinical Supervision Scale | FCP | Digital |
