# Supplementary material for: Cluster randomised feasibility trial of PRISM: the PRimary Care Individual Social Norms MSK Data Dashboard to support first contact physiotherapy management of musculoskeletal patients in primary care
Source: BMJ Open. 2026 Jul 21;16(7):e118099. doi: 10.1136/bmjopen-2026-118099 (PMC13404854; doi:10.1136/bmjopen-2026-118099)
Supplement: online supplemental file 4 [file bmjopen-16-7-s004.docx]

**Consent Form for First Contact Physiotherapist participants in the PRISM Study**

Research Ethics Committee Approval ID Number: **REC reference:** **25/EM/0256**

**Title of Study:**

| **Full title of trial** | PRISM: The PRimary Care Individual Social Norms MSK Data Dashboard: a cluster randomised feasibility trial in First Contact Physiotherapy management of musculoskeletal patients. |
| --- | --- |
| **Short title** | PRISM: The PRimary Care Individual Social Norms MSK Data Dashboard: a feasibility trial |

**Department: Department of Primary Care and Population Health, University College London**

**Name and Contact Details of the Researcher:**

| ***PRINCIPAL RESEARCHER:***  **Dr Emma Dunphy**  **Dept Primary Care & Pop Health**  **Upper Third Floor UCL Medical School (Royal Free Campus) Rowland Hill Street London NW3 2PF**  **emma.dunphy@ucl.ac.uk** | **Prof Irwin Nazareth**  **Dept Primary Care & Pop Health**  **Upper Third Floor UCL Medical School (Royal Free Campus) Rowland Hill Street London NW3 2PF**  **i.nazareth@ucl.ac.uk** | **Prof Jonathan Hill**  **MacKay Building 1.27 / DJW 1.109**  **Keele,**  **Newcastle**  **ST5 5BG**  **j.hill@keele.ac.uk** |
| --- | --- | --- |

Dear First Contact Physiotherapist,

**CONSENT FORM**

1. I confirm that I have read and understood the FCP Information Sheet version ________ for the above study, including the purpose of the study and what my participation involves.
2. I understand that my participation is voluntary and that I am free to withdraw at any time without giving any reason.
3. I understand that relevant sections of data collected during the study may be looked at by authorised individuals from the research team, regulatory authorities, or the sponsor. I give permission for these individuals to access my data.
4. I understand that anonymised data may be shared with other researchers or used in future research, publications, or presentations.
5. I understand that my identity will be protected and that any data published will not contain identifiable information.
6. I understand that my team lead will provide monthly data uploads from my clinical activity as an FCP and I will participate in structured clinical supervision using the PRISM dashboard and guidebook.
7. I understand I can receive a copy of the lay summary of results by post or email.
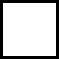

8. I agree to take part in the above study
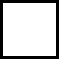


__________________________________________________________________

Name of FCP Date Signature

__________________________________________________________________

Trust/Site of FCP

__________________________________________________________________

Email address of FCP

__________________________________________________________________

Name of Person Date Signature

taking consent

____________________

or Digital Consent Y/N
