# Supplementary material for: Cluster randomised feasibility trial of PRISM: the PRimary Care Individual Social Norms MSK Data Dashboard to support first contact physiotherapy management of musculoskeletal patients in primary care
Source: BMJ Open. 2026 Jul 21;16(7):e118099. doi: 10.1136/bmjopen-2026-118099 (PMC13404854; doi:10.1136/bmjopen-2026-118099)
Supplement: online supplemental file 5 [file bmjopen-16-7-s005.docx]

**Consent Form for the PRISM Study**

Research Ethics Committee Approval ID Number: XXXXX

**Title of Study:**

| **Full title of trial** | PRISM: The PRimary Care Individual Social Norms MSK Data Dashboard: a cluster randomised feasibility trial in First Contact Physiotherapy management of musculoskeletal patients. |
| --- | --- |
| **Short title** | PRISM: The PRimary Care Individual Social Norms MSK Data Dashboard: a feasibility trial |

**Department: Department of Primary Care and Population Health, University College London**

**Name and Contact Details of the Researcher:**

| ***PRINCIPAL RESEARCHER:***  **Dr Emma Dunphy**  **Dept Primary Care & Pop Health**  **Upper Third Floor UCL Medical School (Royal Free Campus) Rowland Hill Street London NW3 2PF**  **emma.dunphy@ucl.ac.uk** | **Prof Irwin Nazareth**  **Dept Primary Care & Pop Health**  **Upper Third Floor UCL Medical School (Royal Free Campus) Rowland Hill Street London NW3 2PF**  **i.nazareth@ucl.ac.uk** | **Prof Jonathan Hill**  **MacKay Building 1.27 / DJW 1.109**  **Keele,**  **Newcastle**  **ST5 5BG**  **j.hill@keele.ac.uk** |
| --- | --- | --- |

Dear First Contact Physiotherapy Service user,

**CONSENT FORM**

Please Initial Box

Please Initial Box

1. I confirm that I have read the participant information sheet dated 21.11.25 (version 3) for the above study. I have had the opportunity to fully consider the information provided and contact the team (contact information on the Patient Information Sheet provided) if I wish to ask questions.
2. I understand that my participation is voluntary and that I am free to withdraw at any

time without giving any reason. This decision will not affect any of my medical care or legal rights being affected.

1. I understand that by agreeing to take part in this study, I consent to completing a health questionnaire and allowing it to be used as a measure of my health and that personal information such as name, age, and ethnicity, will be used solely for research purposes and handled with strict confidentiality in accordance with national data protection regulations and the strict research regulations drawn up by UCL.
2. I understand that the information collected will be used to support other research in the future and may be shared anonymously with other researchers in England within the bounds of the same data regulation laws.
3. (If appropriate) I agree to my General Practitioner being informed of my participation in this study.
4. I understand I can receive a copy of the lay summary of results either by post or email if I agree to take part.
5. I agree to take part in the above study.

Name of Participant Date Signature

__________________________________________________________________

Email address of participant and phone number of participant

Name of Person Date Signature

taking consent

or Digital Consent
