# Supplementary material for: Cluster randomised feasibility trial of PRISM: the PRimary Care Individual Social Norms MSK Data Dashboard to support first contact physiotherapy management of musculoskeletal patients in primary care
Source: BMJ Open. 2026 Jul 21;16(7):e118099. doi: 10.1136/bmjopen-2026-118099 (PMC13404854; doi:10.1136/bmjopen-2026-118099)
Supplement: Supplementary file 6 [file bmjopen-2026-118099.reviewer_comments.pdf]

PEER REVIEW HISTORY

BMJ Open publishes all reviews undertaken for accepted manuscripts. Reviewers are asked to complete a checklist review form and are provided with free text boxes to elaborate on their assessment. These free text comments are reproduced below.

ARTICLE DETAILS

Title (Provisional)

Protocol. A cluster randomised feasibility trial of PRISM: The PRimary Care Individual Social Norms MSK Data Dashboard to support First Contact Physiotherapy management of musculoskeletal patients in Primary Care.

Authors

Dunphy, Emma; Marston, Louise; Hunter, Rachael; Hill, Jonathan C; Downey, Anne M; Forsyth, Sharon; Nwankwo, Chidimma; Nazareth, Irwin

---

VERSION 1 - REVIEW

Reviewer 1

Name Zadro, Joshua

Affiliation University of Sydney, Institute for Musculoskeletal Health, School of Public Health

Date 15-Mar-2026

COI None

I thank the authors for the opportunity to review this interesting and well-designed feasibility trial protocol. The PRISM database is a novel intervention that could improve the use of evidence-based care among FCPs and warrants investigation in a trial before wide-spread implementation. The trial is well-designed with strengths including a comprehensive description of the intervention, recruitment and data collection processes, and a clear rationale. I do have a few queries that should be addressed before this is published.

1) A key rationale for the PRISM dashboard in the introduction was that some FCPs work in isolation and do not have accessing to mentoring/supervision. However, the inclusion criteria only captures FCPs with an experienced physiotherapist supervisor. Please explain the reason behind this inclusion item and the effect it may have on the usefulness of PRISM.

2) There seems to be an assumption that normative behaviour amongst physiotherapists aligns with evidence-based guidelines. However, a lot of work has shown many physiotherapists do not follow guidelines. https://bmjopen.bmj.com/content/9/10/e032329. Does PRISM identify which practices are evidence-based and not? Or does it just list treatment practices which are common/uncommon and encourage FCPs to align their

practices with the majority. If many FCPs are providing non-evidence-based care, there is a risk PRISM would further exacerbate this issue. Further discussion of this issue would be useful.

3) Research question: The current wording makes it sound like you're assessing the feasibility of conducting a feasibility trial. I suggest changing the wording to something more closely aligned to the aims e.g., "Is it feasible to recruit and retain patients and FCPs to the PRISM study? Is there sufficient uptake of (or adherence to?) the PRISM intervention? Is it feasible to collect patient outcomes, experience measures and costs for a future clinical trial? etc.

4) Aim and objectives: make the primary outcome/aim explicit as per the abstract and outcomes section. Ensure the wording of the aim in this section matches exactly the wording in the abstract

5) Study design: consider reporting this study according to the CONSORT extension to randomised pilot and feasibility trials https://www.equator-network.org/reporting-guidelines/consort-2010-statement-extension-to-randomised-pilot-and-feasibility-trials/

6) Please include feasibility targets for key outcomes (e.g., retention, engagement, missing data, drop out) that would support proceeding to a full trial

---

VERSION 1 - AUTHOR RESPONSE

Reviewer: 1
Mr. Joshua Zadro, University of Sydney
Comments to the Author:
I thank the authors for the opportunity to review this interesting and well-designed feasibility trial protocol. The PRISM database is a novel intervention that could improve the use of evidence-based care among FCPs and warrants investigation in a trial before wide-spread implementation. The trial is well-designed with strengths including a comprehensive description of the intervention, recruitment and data collection processes, and a clear rationale. I do have a few queries that should be addressed before this is published.

1) A key rationale for the PRISM dashboard in the introduction was that some FCPs work in isolation and do not have accessing to mentoring/supervision. However, the inclusion criteria only captures FCPs with an experienced physiotherapist supervisor. Please explain the reason behind this inclusion item and the effect it may have on the usefulness of PRISM.

Many thanks for your comment. As a team we did discuss the varied landscape of FCP supervision and how to manage it. Here is my justification for our decisions.

In the background section, I highlighted “FCPs in GP practices work alone (9) and lack the peer support or oversight of a typical physiotherapy department”. This study does not report evidence of FCPs working with no supervision, though anecdotally, this has been suggested to be true. Many

FCPs do have remote supervision on Teams (or similar), the frequency and quality is not well known. Other sites have team/peer learning/training events which are labelled as a supervisory activity.

In constructing the trial, we decided to have supervision as an inclusion criteria as we did not in any way wish to endorse the absence of it. Especially given the evidence in favour of it, that I captured in paragraph 5 of the introduction. The PRISM tool works within the learning environment of clinical supervision and aims to enhance it with data supported reflective practice tools. We also use the Manchester Clinical Supervision Scale to try and capture the details of supervision in both intervention and control groups. This way, when we move forward to trial we will be able to speak clearly about how supervision is conducted and the experiences of FCPs.

2) There seems to be an assumption that normative behaviour amongst physiotherapists aligns with evidence-based guidelines. However, a lot of work has shown many physiotherapists do not follow guidelines. https://bmjopen.bmj.com/content/9/10/e032329. Does PRISM identify which practices are evidence-based and not? Or does it just list treatment practices which are common/uncommon and encourage FCPs to align their practices with the majority. If many FCPs are providing non-evidence-based care, there is a risk PRISM would further exacerbate this issue. Further discussion of this issue would be useful.

Thank you for this important question. Its one we wrestled with, in terms of where to start with thus type of intervention. At this time, we are still learning about how clinical teams collect and submit data, use the dashboard and integrate data into their reflective practice. In future, we want to do more to tie data to evidence based practice and quality care. At present, the social norms process reduces outliers and range of variation. The evidence for this, as illustrated most clearly in the Antibiotic Stewardship programme, does not define, a correct level of antibiotic prescribing, but highlights consistent outliers so that they have the opportunity to know that they are outliers and reflect on whether or not this is suitable.

At present, PRISM reports the data without asserting any quality or judgment based on the numbers. This is explained in every training and engagement session. Norms are not good or bad. They are a reflection of our current practice. Outliers need to be understood and as you suggest may be justifiable and good practice. The guidebook includes examples of how to use the data to move toward quality indicators. For example: a high user of MRI could be running a complex spinal clinic so locally, this makes good sense. The judgment of the data is left to the local team, supervisor and FCP. The PRISM guidebook reminds users of exactly what you have raised and illustrates way to consider the data. It gives examples of the right questions to ask. Ie. If imaging is high, but referral to specialist MSK services/physio is low, why would that be? If age of population is high but referral to Falls clinic are low or zero, is something being missed? If population IMD indicates poverty and heath exclusion factors, then we would expect referrals to personalised care services such as social

prescribing, health and wellbeing, exercise on referral etc. Is this happening and if not, is there a good reason? If smoking or obesity are high in population then we might expect smoking cessation or weight management service referrals as well as MSK interventions. PRISM and guidebook encourage questions to be asked of the data. An upcoming paper on intervention design of PRISM explores this in more detail.

Following this research, once we understand more about how people use PRISM and what the effectiveness of its behaviour change techniques are, we intend to iterate a future version which links clinical activity to 'actionable insights' that are mediated by guidelines and quality indicators. At this time, we are still learning about how clinician teams collect and submit data, use the dashboard and integrate data into their reflective practice.

3) Research question: The current wording makes it sound like you're assessing the feasibility of conducting a feasibility trial. I suggest changing the wording to something more closely aligned to the aims e.g., "Is it feasible to recruit and retain patients and FCPs to the PRISM study? Is there sufficient uptake of (or adherence to?) the PRISM intervention? Is it feasible to collect patient outcomes, experience measures and costs for a future clinical trial? etc.

Thanks for clarity. Have changed to your suggested wording.

4) Aim and objectives: make the primary outcome/aim explicit as per the abstract and outcomes section. Ensure the wording of the aim in this section matches exactly the wording in the abstract

Thank you for catching this. Have used same wording for consistency.

5) Study design: consider reporting this study according to the CONSORT extension to randomised pilot and feasibility trials https://www.equator-network.org/reporting-guidelines/consort-2010-statement-extension-to-randomised-pilot-and-feasibility-trials/

I did in fact use the CONSORT. It was my default having use it before. However, this journal requests Spirit so I changed to that.

6) Please include feasibility targets for key outcomes (e.g., retention, engagement, missing data, drop out) that would support proceeding to a full trial

I am leaning on the MRC guidance as to what is best in this scenario. There is really no evidence of what these benchmarks should be. The pool of FCP evidence in primary care is small and even

smaller when it comes to dashboard/data interventions. It seems we cant therefore have an a priori quantitative benchmark that would be robust and defensible. It might led to ill advised stop/go criteria before we even understand the research landscape we are in. The MRC guidance suggests that feasibility studies are undertaken precisely because key parameters are not known and cant be assumed.

VERSION 2 - REVIEW

**Reviewer**	**1**
**Name**	**Zadro, Joshua**
**Affiliation**	**University of Sydney, Institute for Musculoskeletal Health, School of Public Health**
**Date**	**27-Apr-2026**
**COI**	

I thank the authors for their response and addressing my comments. I do not have anything further to add.
